# Water quality monitoring records for estimating tap water arsenic and nitrate: a validation study

**DOI:** 10.1186/1476-069X-9-4

**Published:** 2010-01-28

**Authors:** Susan Searles Nielsen, Carrie M Kuehn, Beth A Mueller

**Affiliations:** 1 Public Health Sciences Division, Fred Hutchinson Cancer Research Center, 1100 Fairview Avenue North, PO Box 19024, M4-C308, Seattle, Washington, USA; 2 Exponent, Bellevue, Washington, USA; 3 Department of Epidemiology, School of Public Health and Community Medicine, University of Washington, Seattle, Washington, USA

## Abstract

**Background:**

Tap water may be an important source of exposure to arsenic and nitrate. Obtaining and analyzing samples in the context of large studies of health effects can be expensive. As an alternative, studies might estimate contaminant levels in individual homes by using publicly available water quality monitoring records, either alone or in combination with geographic information systems (GIS).

**Methods:**

We examined the validity of records-based methods in Washington State, where arsenic and nitrate contamination is prevalent but generally observed at modest levels. Laboratory analysis of samples from 107 homes (median 0.6 μg/L arsenic, median 0.4 mg/L nitrate as nitrogen) served as our "gold standard." Using Spearman's rho we compared these measures to estimates obtained using only the homes' street addresses and recent and/or historical measures from publicly monitored water sources within specified distances (radii) ranging from one half mile to 10 miles.

**Results:**

Agreement improved as distance decreased, but the proportion of homes for which we could estimate summary measures also decreased. When including all homes, agreement was 0.05-0.24 for arsenic (8 miles), and 0.31-0.33 for nitrate (6 miles). Focusing on the closest source yielded little improvement. Agreement was greatest among homes with private wells. For homes on a water system, agreement improved considerably if we included only sources serving the relevant system (ρ = 0.29 for arsenic, ρ = 0.60 for nitrate).

**Conclusions:**

Historical water quality databases show some promise for categorizing epidemiologic study participants in terms of relative tap water nitrate levels. Nonetheless, such records-based methods must be used with caution, and their use for arsenic may be limited.

## Background

Tap water may be an important source of arsenic and nitrate exposure, but obtaining and analyzing water samples from epidemiologic study participants is expensive and time-consuming. Further, tap water samples may not be available for all participants or reflect levels during the relevant time period, yet inferences about the relationship between disease and exposure are frequently based on present-day contaminant levels from only those who still live at a relevant residence and agree to water testing. Exposure assessment methods that address these shortcomings are needed.

Many developed countries routinely monitor drinking water quality. In the U.S., water purveyors serving > 15 residences or > 25 people have monitored their sources (e.g. wells and rivers) under the federal Safe Drinking Water Act (SDWA) passed in 1974. Currently there are standards for > 90 contaminants. Levels measured in specific water sources may be available and have potential to assign participants of epidemiologic studies into relative exposure categories. Typically the water purveyor or source is determined, and respective records used to estimate contaminant levels in individuals' tap water, alone or in combination with other exposure information. This approach has been frequently used in North America, Europe and Asia, including in recent years for a variety of contaminants and outcomes in adults and children ([1-8]). However, the validity of this approach has been rarely examined (e.g. for nitrate [9]). Furthermore, participants may not know their source of water, especially for their former residences. A residential history or an address at birth or diagnosis may be easier to obtain. Within Washington State we examined whether such methods yield precise exposure estimates for two contaminants (arsenic and nitrate), how accurately residents report their current water purveyor, and the extent to which street address alone can be used to identify the purveyor.

Identifying water purveyor in this manner can be labour intensive, and not all homes receive water from a publicly monitored water system. Therefore, we also explored whether relative tap water levels of these contaminants could be determined by instead using geographic information systems (GIS) methods to link residences to water quality monitoring data. We hypothesized that this method might be feasible because water sources are geographically referenced in these data, and some contaminants are geographically dispersed (i.e. water contamination in a region may reflect levels in the home). We focused on arsenic and nitrate because they tend to be regionally dispersed, are prevalent in our region, are often the focus of etiologic studies of environmental contaminants, and have been regulated since the inception of the SDWA.

One advantage of using public water monitoring data is the possibility of conducting records-based studies. Thus, we also examined the potential for misclassification of exposure due to residential use of bottled water and filtration devices. Prior research indicates this might be important [10].

## Methods

### Participant selection, interview and water collection

Detailed methods have been described [11]. Briefly, we used an added-digit technique [12] to identify, via telephone, a sample of 156 residences with children in regions of Washington State with varying levels of arsenic and nitrate. These homes represented 98% of eligible residences (72% of reached residences were screened). We asked whether the home was on a water system, the purveyor's name, and about use of bottled water and filters. Most (95%) survey participants agreed to provide a tap water sample; 107 (72%) did. Water-sampled homes were more likely than the remainder of surveyed homes to be located within city/town limits (52% vs. 33%) and supplied by a water system (83% vs. 76%), but very similar proportions received groundwater (vs. surface water), and used bottled water and/or filters. In addition, among homes served by a system, the mean, standard deviation, median and range of the mean arsenic and nitrate levels for the respective systems were very similar when comparing homes that did and did not provide a water sample.

Water samples were collected by study participants using a kit we provided. We requested they run the cold tap for 2 minutes prior, circumvent filters, and overnight ship the sample in a cooler with ice packs. The majority of samples (77%) arrived at the study lab (North Creek Analytical, Bothell, WA) the day after collection and at < 6°C. Institutional Review Board approval was received prior to study conduct, and consent was obtained via telephone (survey respondents) and in writing (participants providing water samples).

### Laboratory analysis

Arsenic [13,14] and nitrate and nitrite as nitrogen [13,15] were quantified by inductively coupled plasma-mass spectrometry and ion chromatography respectively, using 250 mL tap water for each analysis. For quality control, residents from 10 additional homes provided a sample, and within 24 hours study staff collected two additional samples from the same home: one for comparison at the study laboratory, and one for testing at the laboratory that certifies others in the state. Excellent agreement was observed between the participant- and staff-collected samples, and between the study and certification laboratory (ρ = 0.89-0.94 for arsenic, ρ = 0.997-1.0 for nitrate, no nitrite).

### Linkage of residences to water purveyor

We assigned a latitude-longitude coordinate to each street address and determined whether the home was located inside city/town boundaries using Maptitude (version 4.1, Caliper Corporation, Newton, MA; 83% geocoded automatically, 17% manually). We also compared each home's coordinate to online water purveyor maps. Some purveyors obtain water from sources managed by other water suppliers, and we used additional online information (mainly from the U.S. Environmental Protection Agency) to determine whether and from whom a purveyor's water was purchased, and whether groundwater or surface water predominantly served each supplier.

### Derivation of summary measures from water monitoring data

The Washington State Department of Health provided water monitoring data for 24,856 drinking water sources throughout the state. These included wells (95%), springs (3%) and surface water sources (2%). Most (86%) were community sources, either monitored under the SDWA (26%) or only under state regulations (fewer than 25 people and 15 connections, 60%). The remainder were non-community sources (e.g. non-residential sources of water consumed by the public). Nearly all (93%) were permanent sources, not emergency or seasonal sources. We included all sources to maximize geographic coverage.

The data also included quantitative laboratory results for arsenic (32,441 samples from the years 1975-2003) and nitrate (82,274 samples from 1975-2003). Testing had occurred at 100 laboratories, and the analytic method(s) used were not specified. We excluded samples unlikely to reflect true values: 11 arsenic and 3 nitrate samples > 10 times the respective federal maximum contaminant level (MCL) and at least an order of magnitude greater than other samples from that water source (presumed to be analytic or recording errors); and 22,219 (68%) arsenic samples reported as 0.01 mg/l while being the most extreme level ever reported for that source (presumed to be artefacts of reporting since this was the MCL at the time, i.e. largely uninformative upper bounds that would introduce substantial "noise").

Records for each water source indicated which supplier received water from it, and the latitude-longitude coordinate of the source itself. Geo-coordinates had been obtained as follows: 22% by global positioning system (GPS), 64% by Bureau of Land Management Public Land Survey System township quarter-quarter-section (0.25 mile × 0.25 mile) centroid, 11% by section (1 mile × 1 mile) centroid, and 4% by other methods. We calculated the distance between each water source and study residence using the haversine great circle distance formula. This equation uses spherical trigonometry to estimate the straight line distance between two latitude-longitude coordinates while accounting for the curvature of the earth. We used water sample data from sources within selected radii ranging from 0.5 to 10 miles to estimate the home's tap water arsenic and nitrate. For each radius and contaminant we calculated the mean; for this measure we present the simple average of all samples in the radius because results were quite similar to those obtained by first averaging the mean contaminant levels in each water source. We also identified the maximum, and the level obtained most recently. We repeated calculations using only samples from the closest water source. Lastly, irrespective of proximity, we calculated the mean using only samples from sources associated with the home's water purveyor (if any). For this measure we conducted subanalyses restricted to samples collected during the same season we collected tap water (+/- one month, regardless of calendar year).

### Statistical analysis

We compared each summary measure to respective contaminant levels in participants' water. Specifically, while retaining these as continuous measures, we estimated precision (hereafter also "agreement"). Because the summary measures we examined likely would be used in lieu of the gold standard (i.e. this was an inter-method comparison), and because contaminant levels were non-normally distributed, we estimated Spearman's correlation coefficient (ρ) as a measure of precision [16]. When ρ = 1.0, the measure to be validated orders all observations perfectly in comparison to the gold standard (i.e. relative exposure levels are fully preserved), and when ρ = 0 there is no relationship between the compared values. We do not report *p*-values because the magnitude of the correlation is of interest: This estimate is useful in the design and interpretation of studies that include the respective exposure measure. For example, when ρ = 0.50, only very strong associations remain detectible [16]. For our results we defined ρ ≤ 0.40 as unacceptable because with this level of precision even strong associations become very difficult to observe.

## Results

### Reporting of water purveyor

Among all surveyed residents of homes on a water system, 74% reported the correct water purveyor. Of those who did not report the correct water purveyor, 39% specified a different type of utility, and another 30% indicated that they rented the home and were unsure. All residents who said they did not have a water purveyor identified the type of private water source and how many homes it supplied. These sources reportedly served no more than eight residences.

### Use of bottled water and filters

Three-quarters of all surveyed homes used some bottled (53%) and/or filtered (38%) water, and 32% used these exclusively. However, only one home (< 1%) had a device particularly well-suited for removing arsenic or nitrate (i.e. a reverse osmosis system). Other types of filters were more common: pitcher (15%), refrigerator (15%), kitchen tap (7%), and whole-house (5%).

### Characteristics of water-sampled residences

Although only 52% of water-sampled homes were within city/town limits, 83% were on a water system, usually operated by a municipality (Table 1). A majority (62%) of on-system homes received groundwater. A private well served each off-system home.

**Table 1:** Characteristics of water-sampled homes, overall and by type of water supply

	All homes N = 107	Public system* N = 89	**Private well**^†^ N = 18
	n (%)	n (%)	n (%)
Within city/town limits	56 (52)	55 (62)	1 (6)
Type of water supply			
Public system	89 (83)	89 (100)	--
Municipality	63 (59)	63 (71)	--
Utility district	12 (11)	12 (13)	--
Private utility/association	14 (13)	14 (16)	--
Private well	18 (17)	--	18 (100)
Shared	7 (7)	--	7 (39)
Individual	11 (10)	--	11 (61)
Water source			
Groundwater	73 (68)	55 (62)	18 (100)
Surface	34 (32)	34 (38)	0 (0)
Arsenic level			
Any detected	97 (91)	82 (92)	15 (83)
> MCL^‡^	0 (0)	0 (0)	0 (0)
	μg/L	μg/L	μg/L
Minimum	0	0	0
25^th ^percentile	0.35	0.35	0.41
Median (μg/L)	0.59	0.56	1.31
75^th ^percentile	1.24	0.97	1.94
Maximum (μg/L)	9.5	4.34	9.5
Nitrate level^§^	n (%)	n (%)	n (%)
Any detected	76 (72)	64 (74)	12 (67)
> MCL^‡^	2 (2)	0 (0)	2 (11)
	mg/L as N	mg/L as N	mg/L as N
Minimum	0	0	0
25^th ^percentile	0	0	0
Median (mg/L)	0.37	0.35	0.39
75^th ^percentile	1.65	1.52	2.27
Maximum (mg/L)	40.5	4.48	40.5
	n (%)	n (%)	n (%)
Nitrite, any detected^§^	0 (0)	0 (0)	0 (0)

* Water system served by wells, surface sources or other sources with federally-mandated monitoring; represented systems served 95 to > 600 000 homes; water came directly or via intermediate purveyors from one of 55 water suppliers, including 12 serving multiple (≤ 12) sampled homes.

† Individual or shared (2-8 homes) private well, not subject to federally-mandated monitoring.

‡ Current U.S. federal maximum contaminant level (10 μg/L arsenic and 10 mg/L nitrate as nitrogen).

§ As nitrogen, among 105 for whom nitrate and nitrite values were determined.

Arsenic and nitrate were detected in most tap water samples (91% and 72%, respectively), but levels were generally far below the MCL (Table 1). No homes on a publicly monitored water system contained arsenic or nitrate near or above the MCL, whereas one private well had 9.5 μg/L arsenic, and two had nitrate above the MCL (18.7 and 40.5 mg/L as nitrogen). Nitrite was not detected.

### Precision of arsenic summary measures

An 8-mile radius was required to link all homes to a publicly monitored water source with usable arsenic data. At this distance, the median number of arsenic samples was 105 (range 1-507) from 1-281 (median 48) water sources. At much shorter radii, the number of residences linking to arsenic-sampled water sources was modest, but precision improved greatly (Table 2). Using a half-mile radius, agreement was acceptable and similar across summary measures (ρ = 0.47-0.51), but only 30% of homes were included. Doubling the radius (1 mile) doubled the homes included (58%), but agreement dropped notably (ρ = 0.26-0.36). Agreement at larger radii was poor to modest (ρ = 0.04-0.32). At radii sufficient to include all homes, use of only the most recent monitoring records maximized agreement.

**Table 2:** Agreement* between arsenic measures, overall and by type of water source

	Radius (maximum distance between residence and water source, miles)										
	0.5	1	2	3	4	5	6	7	8	9	10
**All residences (N = 107)**											
**% with monitoring data in radius**	30	58	78	89	92	93	98	99	100	100	100
Agreement*											
*All sources in radius*											
Mean	0.49	0.30	0.32	0.20	0.18	0.15	0.12	0.04	0.05	0.08	0.09
Maximum	0.51	0.26	0.26	0.21	0.24	0.20	0.16	0.12	0.15	0.16	0.17
Most recent	0.49	0.33	0.13	0.10	0.17	0.07	0.20	0.17	0.24	0.25	0.31
*Closest source only*											
Mean	0.49	0.35	0.19	0.11	0.10	0.09	0.11	0.11	0.13	0.13	0.13
Maximum	0.47	0.36	0.19	0.11	0.10	0.09	0.10	0.10	0.12	0.12	0.12
Most recent	0.49	0.35	0.17	0.09	0.08	0.08	0.09	0.09	0.11	0.11	0.11
											
**Residences on a public system† (N = 89)**											
**% with monitoring data in radius**	31	60	81	89	91	93	98	99	100	100	100
Agreement* (mean, all sources in radius)	0.41	0.21	0.29	0.24	0.22	0.15	0.09	0.02	0.03	0.08	0.09
											
**Residences with a private well‡(N = 18)**											
**% with monitoring data in radius**	22	50	61	89	94	94	100	100	100	100	100
Agreement* (mean, all sources in radius)	1.0	0.68	0.67	0.22	0.32	0.32	0.35	0.25	0.27	0.29	0.32

* Correlation (Spearman's rho) between arsenic levels measured in tap water of individual residences and selected summary measures estimated for that home using public water quality monitoring data and geographic information systems (GIS).

^† ^Water system served by wells, surface sources or other sources with federally-mandated monitoring.

‡ Individual or shared private well, not subject to federally-mandated monitoring.

For most radii, agreement was substantially better for homes off rather than on a water system (Table 2). With an 8-mile radius agreement was 0.27 and 0.03, respectively. At this radius all of the on-system homes linked to at least one source that did not supply the home. Agreement for on-system homes improved markedly (ρ = 0.29) when we used only samples from water source(s) serving the respective water system (not shown). Had we not excluded 0.01 mg/L arsenic reports, agreement would have been only 0.04. When we restricted to records from the same season, we could only include two-thirds of on-system homes, but agreement improved to 0.42.

### Precision of nitrate summary measures

A 6-mile radius was necessary to link all homes to a publicly monitored water source with nitrate data. At this radius, the median number of nitrate samples was 587 (range 3-2,797) from 1-550 (median 127) water sources. For the most part, agreement decreased as the radius increased (Table 3). The summary measure based on mean nitrate in all sources within the respective distance was consistently most strongly correlated with homes' tap water nitrate. However, when agreement was maximized (half-mile radius, ρ = 0.49), only half of homes could be included, and agreement dropped to 0.32 when the radius was sufficient to include all homes.

**Table 3:** Agreement* between nitrate measures, overall and by type of water source

	Radius (maximum distance between residence and water source, miles)										
	0.5	1	2	3	4	5	6	7	8	9	10
**All residences (N = 105)**											
**% with monitoring data in radius**	50	72	87	92	94	98	100	100	100	100	100
Agreement*											
*All sources in radius*											
Mean	0.49	0.44	0.39	0.34	0.34	0.30	0.32	0.32	0.31	0.32	0.34
Maximum	0.37	0.37	0.27	0.26	0.28	0.28	0.33	0.29	0.29	0.29	0.25
Most recent	0.38	0.17	0.31	0.32	0.25	0.27	0.31	0.30	0.28	0.21	0.18
*Closest source only*											
Mean	0.34	0.32	0.25	0.25	0.25	0.24	0.25	0.25	0.25	0.25	0.25
Maximum	0.19	0.26	0.19	0.20	0.19	0.21	0.20	0.20	0.20	0.20	0.20
Most recent	0.35	0.33	0.24	0.24	0.23	0.22	0.21	0.21	0.21	0.21	0.21
											
**Residences on a public system† (N = 87)**											
**% with monitoring** **data in radius**	47	72	87	92	94	99	100	100	100	100	100
Agreement* (mean, all sources in radius)	0.49	0.39	0.35	0.29	0.29	0.24	0.29	0.30	0.28	0.31	0.35
											
**Residences with a private well‡(N = 18)**											
**% with monitoring data in radius**	61	72	83	94	94	94	100	100	100	100	100
Agreement* (mean, all sources in radius)	0.14	0.60	0.40	0.51	0.47	0.44	0.47	0.46	0.43	0.41	0.40

* Correlation (Spearman's rho) between nitrate levels measured in tap water of individual residences and selected summary measures for that home estimated using public water quality monitoring data and geographic information systems (GIS).

^† ^Water system served by wells, surface sources or other sources with federally-mandated monitoring.

‡ Individual or shared private well, not subject to federally-mandated monitoring.

Precision was somewhat better for homes off, rather than on, a water system (Table 3). When using a 6-mile radius, respective agreement was 0.47 and 0.29. For homes on a system, all linked to at least one source that did not supply the home, and agreement was substantially improved by using only records pertaining to the respective water system (ρ = 0.60, not shown). Considering seasonality did not improve agreement.

## Discussion

Our results indicate that publicly available water quality monitoring data might be used to estimate relative levels of some drinking water contaminants for participants in epidemiologic studies, but highlight several important limitations. In general, the approaches examined here worked better for nitrate than arsenic. This may have been due to the number and quality of records available. There were substantially more nitrate than arsenic records, and it was important to exclude arsenic records we believed to be reported as an upper bound. In addition, precision for arsenic slightly improved by using only the most recent records. This may reflect variation in arsenic levels over time, which may occur in our region [17]. Elsewhere [10] correlation between residential tap water arsenic over a much shorter period of time was strong but imperfect (r = 0.88), confirming the plausibility of modest improvements when focusing on water records closest in time to water sampling. However, we expected [18] but did not observe this for nitrate. Thus, alternatively, perhaps more recent samples for arsenic are analyzed or recorded with greater accuracy (the most recent arsenic samples included here followed the announcement of the lower MCL for arsenic, whereas there were no regulatory changes for nitrate). Thus, as new records accumulate, water quality monitoring databases may be increasingly useful for estimating arsenic. At the same time, our observations underscore the possibility that the validity of such methods may vary substantially by contaminant.

For both arsenic and nitrate we developed several summary measures. As expected, there was variation in how well each correctly ordered households with regard to actual levels. More important, however, were the radius (maximum distance) between the home and sampled water source, and whether the home was on a water system. Precision increased as the radius decreased, but ability to link homes to any sampled water source also decreased. This effect was sufficiently pronounced that if agreement became marginally acceptable (ρ > 0.40), "participation" percentages ranged from poor to marginal (30% for arsenic, and 50-72% for nitrate). Precision was also greater for homes off, rather than on a water system. Perhaps for homes with private wells, the spatial relationship with its actual water source is more geographically based than for homes served by a water system. This may be particularly true for those relying on surface sources, which may be quite distant from their ultimate tap destinations. Contaminant levels measured at the source may more likely reflect levels at the tap in homes not on systems, i.e. without an intermediate supplier that may treat and mix water to meet water quality standards. Although the number of homes in our study on private wells was very small, and our GIS-based estimates may have benefited from state-mandated monitoring that more than doubled the number of monitored sources included, it is encouraging that precision appeared greatest for this subset of homes for which it would be impossible to apply the more traditional approach of linking water quality records according to which system served the home.

It is likewise encouraging that for homes on a water system, precision of the simple linkage-by-system approach is good, at least for nitrate. This approach yielded modest agreement for arsenic. However, taking season into account might possibly improve agreement.

Respondents had only moderate knowledge of their water purveyor, but we were able to assign each home to a water purveyor using maps, available online in some regions. Thus, it appears possible that by combining approaches examined here, one could include all or most participants in a study, whether or not they were on a water system or could provide water purveyor information. Use of multiple approaches in one study is not novel, but inclusion of all study participants may reduce the potential for bias, as long as statistical analyses account for the possibility that different exposure assessment methods imply different degrees of measurement error. We assessed one component of this, precision, and observed that it did differ between methods, as well as between homes on and off a water system.

Because precision differed by type of water supply, our overall estimates of precision should be interpreted with care. They may be specific to our region. Previous studies that have explored records-based methods for assessing tap water arsenic or nitrate either focused on homes with private wells [19] or on homes supplied by a system [9], allowing comparison to our water supply-specific results. Our estimates of precision were fairly similar to those reported in these studies, both of which included substantially more homes.

In southeastern Michigan, several spatial models of groundwater arsenic were developed using samples from 6050 private wells, and validated [19] using samples from 371 private wells in a case-control study. A geographic model that secondarily took into account geologic formations and geographic boundaries of bedrock performed best (ρ = 0.46). Models more similar to our basic GIS linkage methods yielded precision closer in magnitude to what we observed for our small subgroup of homes on private wells. One model used mean arsenic within a township (typically 6 × 6 miles; 4.24 miles maximum from the centre), and when we focused on homes served by private wells and used a radius of 4 miles our results were nearly identical (ρ = 0.35 vs. ρ = 0.36). The authors repeated this model using a township section (1 × 1 mile; 0.7 miles maximum from the centre). As in our analysis, precision improved (ρ = 0.42) with a shorter "radius," but data were unavailable for half of homes. Also as we observed, there was little difference between this method and using only samples from the closest well (ρ = 0.35). The similarities between our results are especially interesting given that arsenic levels were greater in that study (median 2.30 μg/L; 90^th ^percentile 22.73 μg/L).

In a German case-control study in which 591 participants lived at a home receiving water from one of 69 public authorities, tap water nitrate was assessed by semi-quantitative test strip and by historical water records [9]. As in Michigan, water contamination levels were greater than in our region (> 50% of controls' tap water exceeded the U.S. MCL of 10 mg/L nitrate as nitrogen). Nonetheless, agreement (ρ = 0.62 for cases, ρ = 0.59 for controls) was nearly identical to our estimate using the most similar method (mean in all water sources supplying the relevant water purveyor, ρ = 0.60). It should be noted, though, that the German study implies greater precision than ours because our "gold standard" (laboratory testing) was presumably better than theirs (test strips). We estimate precision of nitrate test strips to be 0.72 [11].

That the nitrate test strips are more precise than the most precise records-based method we examined deserves discussion. Attenuation of odds ratios (ORs) would be substantial even when using the test strip. An observable OR is a function of the true OR and precision, such that when measurement error does not depend on the outcome, the observable OR per unit increase can be estimated by taking the true odds ratio to the power of the square of precision [16,20]. With a true OR of 2.0, the observable OR when using the nitrate test strip would be 1.43. Use of the best methods examined here for homes and on and off systems (linkage by system and linkage using GIS, respectively) would yield observable ORs of only 1.28 and 1.17. Still, the test strip method relies on subjective comparison of the moistened strip to a colour chart. Bias in ORs due to differential measurement error (including away from the null) might occur if the outcome is already known to the person using the strip [11], whereas linkage-based methods can be applied objectively. Further, for some contaminants, including arsenic [21], a test strip or other in-the-field method suitable for study participants does not exist. Also, it is not always feasible to obtain water from the residence of interest [22], and use of records might allow one to consider past contaminant levels, including those at past residences. The importance of this has been documented [23]. Finally, use of a records-based method might allow a relatively quick and cost-effective study, perhaps without contacting participants. In such case, increasing sample size might be feasible and help compensate for greater measurement error, as well as any added error if one cannot ask participants how much water they consumed, or whether they used bottled water or filters.

In our study, nearly a third of homes with children reported exclusively drinking bottled/filtered water at home. It is likely these practices are even more prevalent in areas with greater levels of contaminants. Failure to take even modest use of filters or bottled water into account during sample size calculations and analysis might substantially impair the ability to detect associations in studies in which the contaminant of interest is removed by the most common types of filters, and for which the main route of exposure is ingestion as opposed to absorption/inhalation during bathing, showering and swimming. For arsenic and nitrate, this might be less problematic if reverse osmosis devices are uncommon, as we observed. Nonetheless, their use should be taken into consideration if possible [10].

Even if such factors are assessed, and the most precise summary measures are employed, studies using the approaches examined here would need to be powered and interpreted in light of the likely effect of an important degree of non-differential measurement error (i.e. noticeably attenuated ORs). Furthermore, water quality monitoring databases may not be suitable for estimating absolute levels of exposure to tap water contaminants. As evidenced by some samples being conservatively reported (i.e. as upper bounds), it may be that these types of records would tend to overestimate absolute contaminant levels. In general this would not be a major limitation for association studies. Precision and "average measurement error" are independent [16]. However, care must be taken in combining these estimates with other sources of exposure (e.g. diet), and in interpreting "cut-points" for categories (e.g. quartiles) as being meaningful.

## Conclusions

Historical water quality databases may be useful in epidemiologic studies that categorize participants by relative levels of tap water nitrate in order to assess the association of this exposure with health outcomes. Such records-based approaches must be applied carefully to avoid introducing bias in ORs or other measures of relative risk. In addition, results must be interpreted with care so that studies that fail to observe an association are not overstated. The use of such methods for arsenic may be limited at present.

## Abbreviations

GIS: geographic information systems; GPS: global positioning system; MCL: maximum contaminant level; μg/L: micrograms per litre; mg/L: milligrams per litre; OR: odds ratio; r: Pearson's correlation coefficient; ρ: Spearman's rho; SDWA: Safe Drinking Water Act.

## Competing interests

The authors declare that they have no competing interests.

## Authors' contributions

BAM conceived the study design, supervised implementation, and contributed to the statistical analysis. SSN helped conceive the study design, conducted statistical analyses, and drafted the manuscript. CMK coordinated the field and laboratory components, and conducted field work and data management. All authors read, edited and approved the final manuscript.

## Authors' information

This work done while Carrie M Kuehn was with the Public Health Sciences Division, Fred Hutchinson Cancer Research Center, Seattle, Washington, USA.
